# The Reaction of Cyanoacetylhydrazine with ω-Bromo(4-methyl)acetophenone: Synthesis of Heterocyclic Derivatives with Antitumor Activity

**DOI:** 10.3390/molecules15053602

**Published:** 2010-05-17

**Authors:** Rafat M. Mohareb, Abeer A. Mohamed

**Affiliations:** 1 Department of Organic Chemistry, Faculty of Pharmacy, October University for Modern Sciences and Arts, October City, A.R., Egypt; 2 Department of Chemistry, Faculty of Science, Cairo University, Giza, A.R., Egypt; 3 National Organization for Drug Control & Research (NODCAR), P.O. 29, Cairo, Egypt; E-Mail: abeer_aam@yahoo.com (A.A.M.)

**Keywords:** hydrazide-hydrazone, 1,3,4-triazine, pyridine, pyridazine, antitumor

## Abstract

New approaches for the synthesis of hydrazide-hydrazone derivatives were demonstrated as well as some heterocyclizations of such derivatives to afford 1,3,4-triazine, pyridine and 1,3,4-oxadiazine derivatives. The antitumor evaluation of the newly synthesized products against three cancer cell lines, namely breast adenocarcinoma (MCF-7), non-small cell lung cancer (NCI-H460) and CNS cancer (SF-268) were recorded. Most of the synthesized compounds showed high inhibitory effects.

## 1. Introduction

Hydrazide-hydrazones are an important class of compounds that has gained much importance in recent years due to their diverse biological activities [1,2,3,4,5,6,7,8,9,10]. The therapeutic prominence of hydrazide-hydrazone derivatives is well established [11,12]. Hydrazide-hydrazones were also reported to elicit anticancer [13,14,15,16,17,18,19,20] and anti-HIV properties [21] and hence they have gained an important place in medicinal chemistry. The discovery of the antineoplastic activity of the naturally occurring Schiff’s bases has stimulated considerable research efforts in the field of condensed systems [22]. With the aim of constructing such condensed systems with the hydrazide-hydrazone nucleus, we turned our attention to using such compounds as synthons for heterocyclic derivatives and their anitumor evaluation [23,24].

## 2. Results and Discussion

**Scheme 1:**
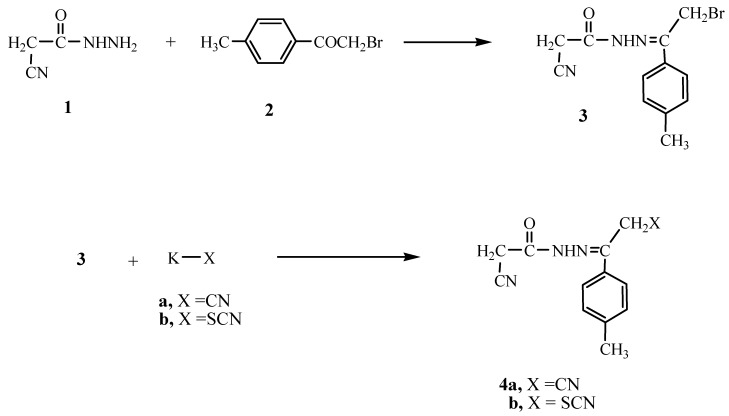
Synthesis of the hydrazide-hydrazone **3** and **4a**, **b**.

In this work we report the reaction of cyanoacetylhydrazine (**1**) with ω-bromo-(4-methyl-acetophenone) (**2**) in 1,4-dioxane which gave the condensed product **3**. The structure of compound **3** was confirmed based on analytical and spectral data. Thus, the ^1^H-NMR showed a singlet at δ 2.51 for the CH_3_, two singlets at δ 4.31, 4.72 for the two CH_2_ groups, a multiplet at δ 6.50–7.76 for the C_6_H_4_ group and a singlet at δ 11.46 (D_2_O exchangeable) for the NH group. The reactivity of compound **3** towards different chemical reagents was studied. The reaction of **3** with either potassium cyanide or potassium thiocyanate gave the corresponding cyanide or the thiocyanate derivatives **4a** and **4b**, respectively (Scheme 1).

**Scheme 2:**
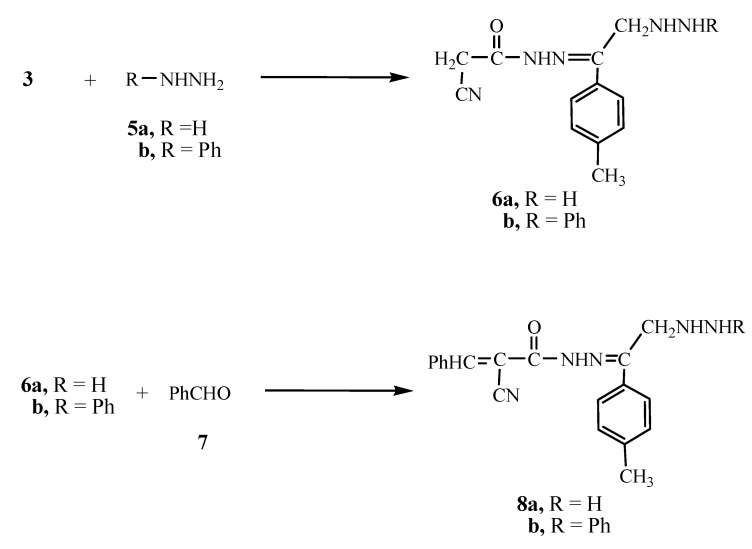
Synthesis of the hydrazine derivatives **6a**, **b** and **8a**, **b**.

The reaction of compound **3** with either hydrazine hydrate (**5a**) or phenylhydrazine (**5b**) gave the hydrazine derivative **6a** or **6b**, respectively. Analytical and spectral data of the reaction products are in agreement with the proposed structures (see Experimental section). The reaction of either **6a** or **6b** with benzaldehyde (**7**) gave the benzal derivative **8a** or **8b**, respectively (Scheme 2).

**Scheme 3:**
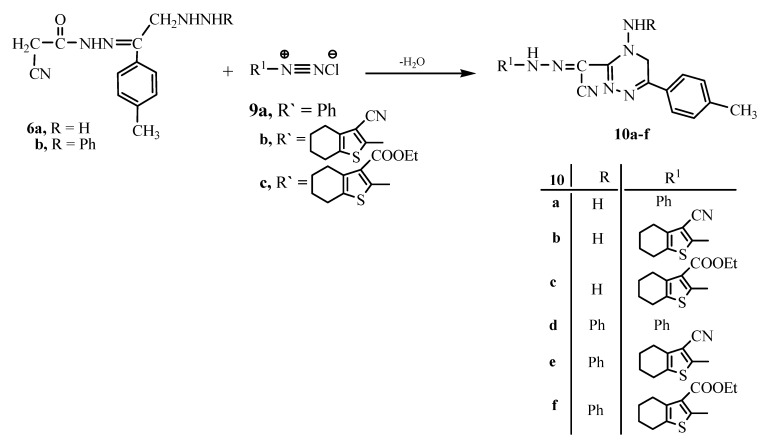
Synthesis of the 1,2,4-triazines **10a–f**.

On the other hand, the reaction of either **6a** or **6b** with either benzenediazonium chloride (**9a**), 3-cyano-4,5,6,7-tetrahydrobenzo[b]thiophene-2-diazonium chloride (**9b**) or ethyl 3-cyano-4,5,6,7-tetrahydrobenzo[b]thiophene-3-carboxylate-2-diazonium chloride (**9c**) gave the 3-(α-hydrazo-acetonitrilo)-1,2,4-triazine derivatives **10a–f**, respectively (Scheme 3). The analytical and spectral data of the latter reaction products are all consistent with the proposed structures.

**Scheme 4:**
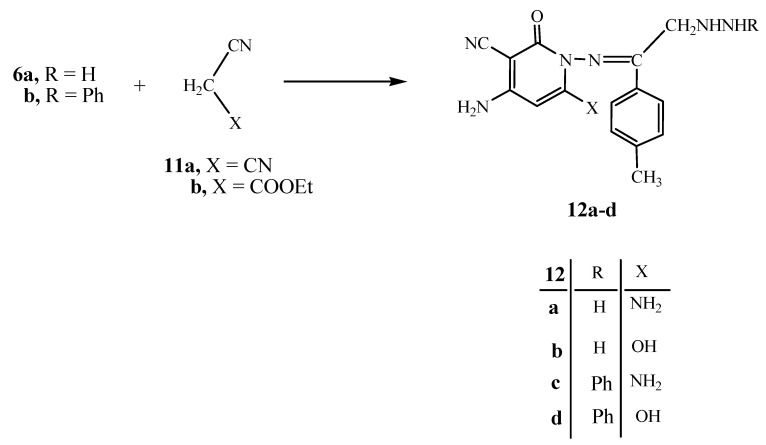
Synthesis of 2-oxopyridines **12a–d**.

Next, we moved towards studying the reactivity of **6a** and **6b** with active methylene reagents. Thus, their reactions with either malononitrile (**11a**) or ethyl cyanoacetate (**11b**) gave the pyridine derivatives **12a–d**, respectively (Scheme 4). The structures of the latter products were established on the basis of their analytical and spectral data. Thus, the ^1^H-NMR spectrum of **12c **showed a singlet at δ 2.51 for the CH_3_ group, a singlet at δ 3.38 for the CH_2_ group, two singlets at δ 4.38, 4.79 for the two NH_2_ groups and a multiplet at δ 6.48–8.19 for the pyridine H-3, C_6_H_5 _and C_6_H_4_, two singlets at δ 10.82, 11.20 for the two NH groups, respectively. 

**Scheme 5:**
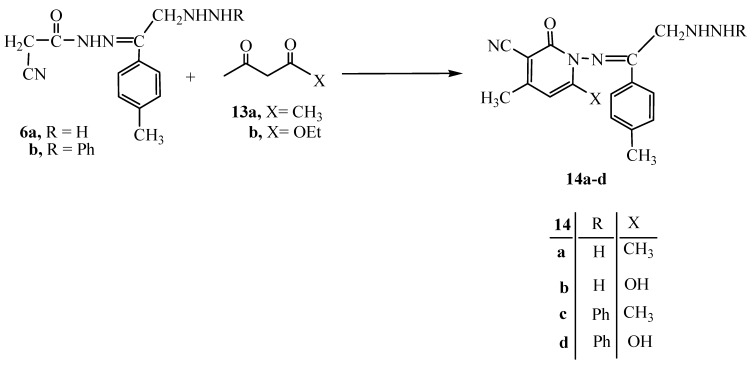
Synthesis of 2-oxopyridines **14a–d**.

On the other hand, the reaction of either **6a** or **6b** with either acetylacetone (**13a**) or ethyl acetoacetate (**13b**) gave the pyridine derivatives **14a–d**, respectively (Scheme 5). The structures of the latter products were confirmed by their analytical and spectral data (see Experimental section). 

**Scheme 6:**
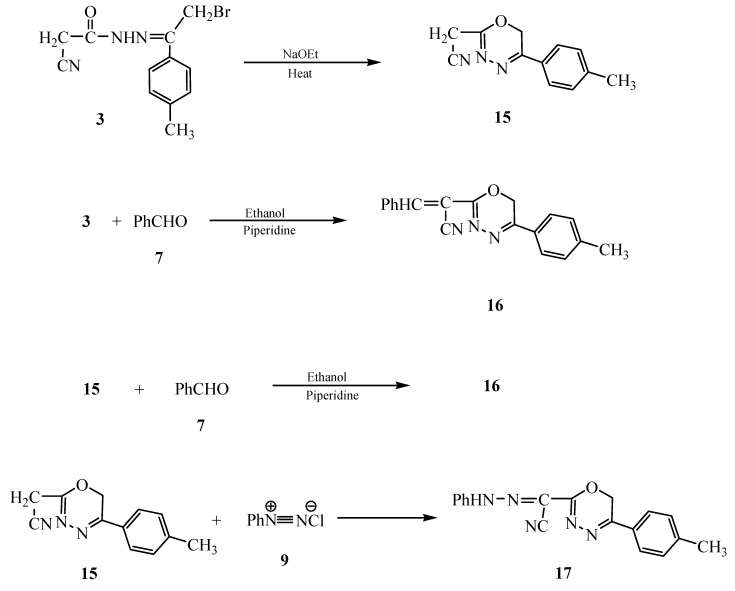
Synthesis of 1,3,4-oxadiazines **15**, **16** and **17**.

Compound **3** underwent ready cyclization when heated in sodium ethoxide solution to give the 1,3,4-oxadiazine derivative **15**, whose structure was established from its analytical and spectral data (see Experimental section). The oxadiazine derivatives **15 **seemed to be an intermediate for many reactions between **3** and many chemical reagents. Thus, the reaction of **3** with benzaldehyde (**7**) gave the 2-(α-benzalacetonitrilo)-1,3,4-oxadiazine derivative **16**. The analytical and spectral data of **16** were in agreement with the proposed structure. Thus, the ^1^H-NMR spectrum showed a singlet at δ 2.51 for the CH_3_ group, a singlet at δ 4.22 for the CH_2_ group, a singlet at δ 5.16 for the (=CH) group and a multiplet at δ 7.35–8.02 for the C_6_H_5 _and C_6_H_4 _groups. The same product **16** was obtained through the reaction of compound **15** with benzaldehyde (**7**) (confirmed by m.p., mixed m.p. and fingerprint IR spectrum). On the other hand, the reaction of **15** with benzenediazonium chloride (**9**) gave the 2-(α-phenylhydrazo)-1,3,4-oxadiazine derivative **17 **(Scheme 6). 

### Effect on the Growth of Human Tumor Cell Lines

The effect of compounds **4**–**17** was evaluated on the *in vitro* growth of three human tumor cell lines representing different tumor types, namely, breast adenocarcinoma (MCF-7), non-small cell lung cancer (NCI-H460) and CNS cancer (SF-268), after a continuous exposure of 48 h. The results are summarized in Table 1. All the compounds were able to inhibit the growth of the tested human tumor cell lines in a dose-dependent manner (data not shown). The results showed that compound **14b**, with its 2-hydroxypyridine group, showed the highest inhibitory effect against all the three tumor cell lines. In addition compounds **10a**, **10f**, **12a**, **12b** and **14c** showed high inhibitory effects. On the other hand, compounds **4b**, **6a**, **8b**, **10b**, **10d**, **12c**, **12d**, **14a**, **14c**, **16** and **17** showed the lowest inhibitory effect towards adenocarcinoma (MCF-7). The rest of compounds showed the moderate growth inhibitory effects. Comparing compound **4a** with **4b**, it is obvious that the presence of the α-SCN present in **4b** showed lower inhibitory effect than **4a** with it’s α-CN group. Comparing 1,2,4-triazine derivatives **10a** (with the 4-phenylamino group) and **10b** (with the 4-thiophenoamino group), the first has a greater inhibitory effect than the second towards the three cell lines.

**Table 1:** Effect of the newly synthesized products on the growth of three human tumor cell lines.

Compound	GI_50_(μΜ)		
	MCF-7	NCI-H460	SF-268
**4a**	10.0 ± 0.6	12.9 ± 1.6	15 ± 1.1
**4b**	70.6 ± 15.3	38.1 ± 10.8	48.0 ± 9.1
**6a**	50.8 ± 18.5	20.2 ± 12.6	50.0 ± 8.7
**6b**	25.8 ± 12.5	22.4 ± 8.6	20.3 ± 8.6
**8a**	24.6*	77.9 ± 5.0	35.0*
**8b**	80.9 ± 10.2	70.9 ± 6.1	50.2 ± 2.2
**10a**	8.0 ± 0.6	11.7 ± 8.8	14.8 ± 1.6
**10b**	70.6 ± 8.6	55.6 ± 5.8	42.6 ± 8.6
**10c**	24.2 ± 0.6	8.4 ± 1.8	10.4 ± 8.4
**10d**	88.4 ± 4.3	66.8 ± 12.0	40.9 ± 2.6
**10e**	12.4 ± 2.6	6.8 ± 1.2	8.3 ± 9.2
**10f**	10.0 ± 0.4	14.9 ± 1.6	14 ± 1.1
**12a**	12.4 ± 1.6	10.8 ± 4.0	16 ± 6.0
**12b**	8.6 ± 2.4	10.4 ± 6.2	12.0 ± 4.2
**12c**	86.8 ± 6.0	48.5 ± 4.0	38.4 ± 2.6
**12d**	90.8 ± 2.4	78.2 ± 2.2	86.2 ± 1.8
**14a**	66.4 ± 6.0	42.4 ± 6.0	62.3 ± 6.0
**14b**	4.2 ± 2.8	10.2 ± 6.8	10.8 ± 5.0
**14c**	80.3 ± 4.0	64.8 ± 6.4	50.8 ± 6.4
**16**	40.5 ± 2.6	32.6 ± 4.0	26.8 ± 8.4
**17**	60.4 ± 6.0	77.8 ± 3.1	47.0 ± 6.4

Results are given in concentrations that were able to cause 50% of cell growth inhibition (GI_50_) after a continuous exposure of 48 h and show means ± SEM of three-independent experiments performed in duplicate. ^*^Results from two-independent experiments performed in duplicate. Doxorubicin was used as positive control, GI_50_: MCF-7 = 42.8 ± 8.2 nM, NCI-H460 = 94.0 ± 8.7 nM, and SF-268 = 94.0 ± 7.0 nM.

## 3. Experimental

### 3.1. General

Melting points were determined on an Electrothermal melting point apparatus (Electrothermal 9100) and are uncorrected. IR spectra were recorded for KBr discs on a Pye Unicam SP-1000 spectrophotometer. ^1^H-NMR & ^13^C-NMR spectra were measured on a Varian EM-390-200 MHz in CD_3_SOCD_3_ as solvent using TMS as internal standard, and chemical shifts are expressed as δ. Analytical data were obtained from the Microanalytical Data Unit at Cairo University, Giza, Egypt. Antitumor evaluation for the newly synthesized products were performed by a research group at the National Research Center & the National Cancer Institute at Cairo University.

*4-Methyl-ω-bromoacetophenone cyanoacetylhydrazone *(**3**)*.* To a solution of cyanoacetylhydrazine (**1**, 2.44 g, 0.02 mol) in 1,4-dioxane (20 mL), ω-bromo-(4-methylacetophenone) (5.24 g, 0.02 mol) was added. The reaction mixture was stirred at room temperature for 1 hr then poured onto a beaker containing an ice/water mixture. The solid product formed was collected by filtration and dried obtaining pale yellow crystals (from ethanol).Yield: 5.02 g (71%), m.p. 148 °C; IR (KBr) υ/cm^−1^: 3400–3378 (NH), 3105 (CH aromatic), 2956 (CH_3_), 2259 (CN), 1681 (C=O), 1610 (C=C);^ 1^H-NMR δ: 2.51 (s, 3H, CH_3_), 4.31, 4.72 (2s, 4H, CH_2_), 6.50–7.76 (m, 4H, C_6_H_4_), 11.46 (s, 1H, NH). MS: (m/z) 293 (M^.+^) (6%), 200 (28%), 117 (100%), 68 (69.9%); ^13^C-NMR: 22.6 (CH_3_), 27.0, 58.7 (2CH_2_), 118.5 (CN), 126.8, 128.4, 129.5, 138.8 (C_6_H_5_), 156.3 (C=N), 173.8 (C=O); *Anal.* Calcd. for C_12_H_12_N_3_OBr (294.24): C, 48.00; H, 4.11; N, 14.29%. Found: C, 49.27; H, 4.45; N, 14.35%.

### 3.2. General Procedure for the Synthesis of ***4a*** or ***4b***

To a solution of **3** (0.54 g, 1.83 × 10^−3^ mol) in ethanol (25 mL) in a water bath at 60 °C, either potassium cyanide (0.11 g, 1.83 × 10^−3^ mol) or potassium thiocyanate (0.17 g, 1.83 × 10^−3^ mol) was added with continuous stirring. The reaction mixture was left in the water bath for 30 min at 60 °C then poured onto a beaker containing ice/water mixture and few drops of hydrochloric acid. The formed solid product was collected by filtration and dried.

*4-Methyl-ω-cyanoacetophenonecyanoacetylhydrazone *(**4a**). Pale brown crystals (from ethanol).Yield: 0.20 g (62%), m.p. 160 °C; IR (KBr) υ/cm^−1^: 3450–3205 (NH), 3031 (CH aromatic), 2260, 2209 (2CN), 1680 (C=O); ^1^H NMR δ: 2.39 (s, 3H, CH_3_), 4.46, 5.06 (2s, 4H, 2CH_2_), 7.18–7.84 (m, 4H, C_6_H_4_), 10.82 (s, 1H, NH); ^13^C NMR: 23.8 (CH_3_), 27.3, 29.5 (2CH_2_), 116.9, 117.0 (2CN), 127.3, 128.0, 129.8, 138.9 (C_6_H_4_), 155.8 (C=N), 173.8 (C=O); *Anal.* Calcd. for C_13_H_12_N_4_O (240.26): C, 64.99; H, 5.03; N, 23.32%. Found: C, 64.79; H, 5.11; N, 22.99%.

*4-Methyl-ω-thiocyanoacetophenoecyanoacetylhydrazone *(**4b**). Buff crystals (from ethanol). Yield: 0.33 g (67%), m.p. 130 °C; IR (KBr) υ/cm^−1^: 3445–3225 (NH), 3099 (CH aromatic), 3034 (CH_3_), 2969, 2925 (2CH_2_), 2264, 2225 (2CN), 1666 (C=O), 1606 (C=C); ^1^H-NMR δ: 2.49 (s, 3H, CH_3_), 4.50, 5.08 (2s, 4H, 2CH_2_), 7.06–7.93 (m, 4H, C_6_H_4_), 11.52 (s, 1H, NH); ^13^C-NMR: 23.8 (CH_3_), 27.3, 29.4 (2CH_2_), 116.7, 117.8 (2CN), 127.0, 128.3, 129.6, 138.6 (C_6_H_4_), 155.5 C=N), 173.9 (C=O); *Anal.* Calcd for C_13_H_12_N_4_OS (272.32): C, 57.33; H, 4.44; N, 20.57; S, 11.77%. Found: C, 57.38; H, 4.64; N, 20.68; S, 11.57%.

### 3.3. General Procedure for the Synthesis of ***6a*** and ***6b***

To a solution of compound **3** (1.50 g, 5.09 × 10^−3^ mol) in ethanol (35 mL) either hydrazine hydrate (0.25 g, 5.09 × 10^−3^ mol) or phenylhydrazine (0.55 g, 5.09 × 10^−3^ mol) was added. The reaction mixture was heated under reflux for 3 hrs then poured onto a beaker containing an ice/water mixture and a few drops of hydrochloric acid. The formed solid product was collected by filtration and dried.

*4-Methyl-ω-hydrazinoacetophenonecyanoacetylhydrazone *(**6a**). Greenish brown crystals (from ethanol). Yield: 0.65 g (52%), m.p. 120 °C; IR (KBr) υ/cm^−1^: 3400–3204 (NH_2_, 2NH), 3027 (CH aromatic), 2918 (CH_3_), 2203 (CN), 1688 (C=O), 1607 (C=C); ^1^H-NMR δ: 2.51 (s, 3H, CH_3_), 3.39, 4.23 (2s, 4H, 2CH_2_), 4.82 (s, 2H, NH_2_), 6.87–8.40 (m, 4H, C_6_H_4_), 9.26, 11.41 (2s, 2H, 2NH); ^13^C-NMR: 23.6 (CH_3_), 27.0, 29.6 (2CH_2_), 127.8, 128.3, 129.5, 138.6 (C_6_H_4_), 155.6, 164.2 (2C=N), 173.8 (C=O); *Anal.* Calcd. for C_12_H_15_N_5_O (245.28): C, 58.76; H, 6.16; N, 28.55%. Found: C, 58.55; H, 5.96; N, 28.32%.

*4-Methyl-ω-phenylhydrazinoacetophenonecyanoacetylhydrazone *(**6b**)*.* Brown crystals (from ethanol). Yield: 0.95 g (63%), m.p. 72 °C; IR (KBr) υ/cm^−1^: 3500–3174 (3NH), 3026 (CH aromatic), 2969 (CH_3_), 2205 (CN), 1687 (C=O), 1600 (C=C); ^1^H-NMR δ: 2.51 (s, 3H, CH_3_), 3.39, 4.35 (2s, 4H, 2CH_2_), 6.79–8.40 (m, 9H, C_6_H_5_, C_6_H_4_), 10.82, 11.19, 12.63 (3s, 3H, 3NH); ^13^C-NMR: 23.6 (CH_3_), 27.0, 29.9 (2CH_2_), 120.3, 122.5, 127.3, 128.4, 129.3, 138.5, 140.1 (C_6_H_4_, C_6_H_5_), 155.6, 164.2 (2C=N), 173.8 (C=O); *Anal.* Calcd. for C_18_H_19_N_5_O (321.38): C, 67.27; H, 5.96; N, 21.79%. Found: C, 67.37; H, 5.84; N, 22.61%.

*α-Benzal-4-methyl-ω-hydrazinoacetophenonecyanoacetylhydrazone *(**8a**)*: *To a solution of compound **6a **(0.29 g, 1.18 × 10^−3^ mol) in ethanol (25 mL) containing piperidine (0.5 mL), benzaldehyde (0.11 g, 1.48 × 10^−3^ mol) was added. The reaction mixture was heated under reflux for 3hrs then poured onto a beaker containing an ice/water mixture and few drops of hydrochloric acid. The formed solid product was collected by filtration and dried obtaining deep yellowish brown crystals (from ethanol).Yield: 0.26 g (66%), m.p. 90 °C; IR (KBr) υ/cm^−1^: 3500–3194 (NH_2_, 2NH), 3027 (CH aromatic), 2919 (CH_3_), 2218 (CN), 1680 (C=O), 1608 (C=C); ^1^H-NMR δ: 2.51 (s, 3H, CH_3_), 3.36 (s, 2H, CH_2_), 4.77 (s, 2H, NH_2_), 5.99 (s, 1H, =CH), 7.27–8.40 (m, 9H, C_6_H_5_, C_6_H_4_), 8.81, 9.15 (2s, 2H, 2NH); ^13^C-NMR: 23.2 (CH_3_), 27.4, 29.2 (2CH_2_), 122.3, 122.8, 122.5, 126.0 128.3, 129.9, 138.3 (C_6_H_5_, C_6_H_4_) 155.0, 164.3 (2C=N), 174.3 (C=O); *Anal.* Calcd. for C_19_H_19_N_5_O (333.39): C, 68.45; H, 5.74; N, 21.01%. Found: C, 68.55; H, 5.54; N, 21.31%.

*α-Benzal-4-methyl-ω-phenylhydrazinoacetophenonecyanoacetyl-hydrazone *(**8b**)**. **To a solution of compound **6b **(0.36 g, 1.12 × 10^−3^ mol) in ethanol (25 mL) containing piperidine (0.5 mL), benzaldehyde (0.11 g, 1.12 × 10^−3^ mol) was added. The reaction mixture was heated under reflux for 3hrs then poured onto a beaker containing an ice/water mixture and a few drops of hydrochloric acid. The formed solid product was collected by filtration and dried obtaining yellowish brown crystals (from ethanol). Yield: 0.33 g (73%), m.p. 102 °C; IR (KBr) υ/cm^−1^: 3300-3179 (3NH), 3026 (CH aromatic), 2920 (CH_3_), 2210 (CN), 1688 (C=O), 1600 (C=C); ^1^H NMR δ: 2.88 (s, 3H, CH_3_), 3.37 (s, 2H, CH_2_), 6.90–8.40 (m, 15H, =CH, 2C_6_H_5_, C_6_H_4_), 9.20, 10.83, 12.62 (3s, 3H, 3NH); ^13^C-NMR: 23.2 (CH_3_), 27.4, 29.2 (2CH_2_), 120.9, 121.1, 122.3, 122.8, 123.4, 123.9, 122.5, 126.0 128.3, 129.5, 138.6 (2C_6_H_5_, C_6_H_4_) 155.3, 164.0 (2C=N), 174.6 (C=O); *Anal.* Calcd. for C_25_H_23_N_5_O (409.48): C, 73.33; H, 5.66; N, 17.10%. Found: C, 73.52; H, 5.97; N, 17.33%.

*1-Amino-6-(4-methylphenyl)-3-(α-phenylhydrazoacetonitrilo)-1,2,4-triazine *(**10a**)**. **To a cold solution (0–5 °C) of compound **6a **(0.40 g, 1.63 × 10^−3^ mol) in ethanol (50 mL) containing sodium hydroxide (10 mL, 10%) and a solution of benzenediazonium chloride (1.63 × 10^−3^ mol) [which was prepared by dissolving sodium nitrite (0.16 g, 2.44 × 10^−3^ mol) in water, 2 mL was added to a cold solution of aniline (0.15 g, 1.63 × 10^−3^ mol) containing the appropriate amount of hydrochloric acid and with continuous stirring] was added with continuous stirring. The formed solid product was collected by filtration and dried to give reddish brown crystals (from ethanol and few drops of dimethylformamide). Yield: 0.58 g (63%), m.p. 170 °C; IR (KBr) υ/cm^−1^: 3400–3307 (NH_2_, NH), 3027 (CH aromatic), 2919, 2862 (CH_3_, CH_2_), 2211 (CN), 1600 (C=C); ^1^H-NMR δ: 2.51 (s, 3H, CH_3_), 3.37 (s, 2H, CH_2_), 4.22 (s, 2H, NH_2_), 6.50–8.20 (m, 9H, C_6_H_5_, C_6_H_4_), 11.64 (s, 1H, NH); ^13^C-NMR: 23.8 (CH_3_), 51.9 (triazine CH_2_), 115.8 (CN), 118.3, 118.9, 119.2, 121.6, 124.8, 133.0, 139.6 (C_6_H_5_, C_6_H_4_), 156.9, 163.8 164.1 (3 C=N); *Anal.* Calcd. for C_18_H_17_N_7_ (331.38): C, 65.24; H, 5.17; N, 29.58%. Found: C, 65.42; H, 5.27; N, 29.36%.

### 3.4. General Procedure for the Synthesis of ***10b*** and ***10c***

To a cold solution (0–5 °C) of compound **6a **(0.49 g, 1.99 × 10^−3^ mol) in ethanol (50 mL) containing sodium hydroxide solution (10 mL, 10%) was added with continuous stirring a solution of either 3-cyano-4,5,6,7-terahydrobenzo[b]thiophene-2-diazonium chloride (**9b**) (1.99 × 10^−3^ mol) or ethyl 4,5,6,7-tetrahydrobenzo[b]thiophene-3-carboxylate-2-diazonium chloride (**9c**) (1.99 × 10^−3^ mol) [which was prepared by dissolving sodium nitrite (0.20 g, 2.99 × 10^−3^ mol) in water, 2 mL was added to a cold solution of either the 2-amino-3-cyano-4,5,6,7-tetrahydrobenzo[b]thiophene (0.35 g, 1.99 × 10^−3^ mol) or ethyl 2-amino-4,5,6,7-tetrahydrobenzo[b]thiophene-3-carboxylate (0.45 g, 1.99 × 10^−3^ mol) dissolved in acetic acid (50 mL) containing the appropriate amount of hydrochloric acid and with continuous stirring]. The solid product formed was collected by filtration and dried.

*1-Amino-6-(4-methylphenyl)-3-(α-(3-cyano-2-hydrazo-4,5,6,7-tetra-hydrobenzo[b]thiophene)aceto-nitrilo)-1,2,4-triazine *(**10b**). Deep brown crystals (from ethanol). Yield: 0.67 g (75%), m.p. 210–214 °C; IR (KBr) υ/cm^−1^: 3600–3425 (NH_2_, NH), 3030 (CH aromatic), 2930 (CH_3_), 2250, 2217 (2CN), 1606 (C=C); ^1^H-NMR δ: 1.63–2.34 (m, 8H, cyclohexene 4CH_2_), 2.51 (s, 3H, CH_3_), 3.60 (s, 2H, CH_2_), 4.78 (s, 2H, NH_2_), 6.97–8.32 (m, 4H, C_6_H_4_), 9.84 (s, 1H, NH); ^13^C-NMR: 19.4, 23.0, 25.1, 27.8 (cyclohexene 4CH_2_), 24.8 (CH_3_), 51.9 (triazine CH_2_), 115.8, 116.4 (2CN), 118.3, 118.9, 119.2, 121.6, 124.6, 133.2, 136.5, 136.9 139.6 (thiophene C, C_6_H_5_, C_6_H_4_), 156.0, 163.5 164.3 (3 C=N); *Anal.* Calcd. for C_21_H_20_N_8_S (416.50): C, 60.56; H, 4.84; N, 26.90; S, 7.70%. Found: C, 60.28; H, 5.03; N, 26.61; S, 7.88%.

*Ethyl 1-amino-6-(4-methylphenyl)-3-(α(2-hydrazo-4,5,6,7-tetra-hydrobenzo[b]thiophene-3-carboxyl-ate)acetonitrilo)-1,2,4-triazine *(**10c**). Brown crystals (from ethanol) Yield: 0.53 g (57%)**,** m.p. 90 °C; IR (KBr) υ/cm^−1^: 3400–3287 (NH_2_, NH), 2976, 2934, 2860 (2CH_3_, CH_2_), 2213 (CN), 1711 (C=O); ^1^H- NMR δ: 1.07–1.92 (m, 8H, cyclohexene 4CH_2_), 2.51 (s, 3H, CH_3_), 2.77 (t, 3H, J = 7.04 Hz, CH_3_), 3.36 (s, 2H, CH_2_), 4.24 (q, 2H, J = 7.04 Hz, CH_2_), 4.99 (s, 2H, NH_2_), 6.50–8.08 (m, 4H, C_6_H_4_), 9.88(s, 1H, NH); ^13^C-NMR: 14.5, 24.6 (2 CH_3_), 23.2, 23.6, 25.1, 27.6 (cyclohexene 4CH_2_), 55.4 (triazine CH_2_), 60.8 (ester CH_2_), 128.7, 129.0, 129.7, 132.1, 136.5, 138.9, 140.8 (C_6_H_5_, thiophene C), 115.9, 116.7 (2CN), 155.2, 158.0, 163.1 (3 C=N), 165.6 (CO); Anal. Calcd. for C_23_H_25_N_7_O_2_S (463.56): C, 59.59; H, 5.44; N, 21.15; S, 6.93%. Found: C, 59.83; H, 5.46; N, 20.91; S, 7.05%.

*1-Phenylamino-6-(4-methylphenyl)-3-(α-phenylhydrazoacetonitrilo)-1,2,4-triazine *(**10d**)*.* To a cold solution (0–5 °C) of compound **6b **(0.53 g, 1.64 × 10^−3^ mol) in ethanol (50 mL) containing sodium hydroxide (10 mL, 10%) was added with continuous stirring a solution of benzenediazonium chloride (1.64 × 10^−3^ mol) [which was prepared by dissolving sodium nitrite (0.17 g, 2.47 × 10^−3 ^mol) in water, 2 mL was added to a cold solution of aniline (0.15 g, 1.64 × 10^−3^ mol) containing the appropriate amount of hydrochloric acid and with continuous stirring]. The formed solid product was collected by filtration to give reddish brown crystals (from ethanol and few drops of dimethylformamide). Yield: 0.58 g (86%), m.p. 120 °C; IR (KBr) υ/cm^−1^: 3500–3422 (2NH), 3057 (CH aromatic), 3028, 2921(CH_3_, CH_2_), 2211 (CN), 1600 (C=C); ^1^H-NMR δ: 2.50 (s, 3H, CH_3_), 3.31 (s, 2H, CH_2_), 6.60–7.65 (m, 14H, 2C_6_H_5_, C_6_H_4_), 8.16, 9.05 (s, 2H, 2NH); ^13^C-NMR: 23.6 (CH_3_), 51.9 (triazine CH_2_), 115.6 (CN), 118.6, 118.7, 119.2, 120.1, 121.6, 124.8, 133.6, 134.8, 139.9 (2C_6_H_5_, C_6_H_4_), 156.6, 163.7, 164.0 (3 C=N); *Anal.* Calcd. for C_24_H_21_N_7_ (407.47): C, 70.74; H, 5.19; N, 24.06%. Found: C, 71.05; H, 5.38; N, 23.87%. 

### 3.5. General Procedure for the Synthesis of ***10e*** and ***10f***

To a cold solution (0–5 °C) of compound **6b **(0.40 g, 1.24 × 10^−3^ mol) in ethanol (50 mL) containing sodium hydroxide solution (10 mL, 10%) was added with continuous stirring a solution of either 3-cyano-4,5,6,7-terahydrobenzo[b]thiophene-2-diazonium chloride **(9b)** (1.24 × 10^−3^ mol) or ethyl 4,5,6,7-tetrahydrobenzo[b]thiophen-3-carboxylate-2-diazonium chloride **(9c)** (1.24 × 10^−3^ mol) [which was prepared by dissolving sodium nitrite (0.12 g, 1.86 × 10^−3^ mol) in water, 2 mL was added to a cold solution of either the 2-amino-3-cyano-4,5,6,7-tetrahydrobenzo[b]thiophene (0.22 g, 1.99 × 10^−3^ mol) or ethyl 2-amino-4,5,6,7-tetrahydrobenzo[b]thiophene-3-carboxylate (0.28 g, 1.99 × 10^−3^ mol) dissolved in acetic acid (50 mL) containing the appropriate amount of hydrochloric acid and with continuous stirring]. The solid product formed was collected by filtration and dried.

*1-Phenylamino-6-(4-methylphenyl)-3-(α-(3-cyano-2-hydrazo-4,5,6,7-tetrahydrobenzo[b]thiophene)-acetonitrilo)-1,2,4-triazine *(**10e**). Reddish brown crystals (from ethanol), yield: 0.34 g (55%), m.p. 190 °C; IR (KBr) υ/cm^−1^: 3500–3425 (2NH), 3030 (CH aromatic), 2930 (CH_3_), 2250, 2217 (2CN), 1606 (C=C); ^1^H-NMR δ: 1.63–2.34 (m, 8H, cyclohexene), 2.51 (s, 3H, CH_3_), 3.60 (s, 2H, CH_2_), 6.97–8.32 (m, 9H, C_6_H_5_, C_6_H_4_), 9.84, 10.00 (s, 2H, 2NH); ^13^C-NMR: 19.1, 23.3, 25.4, 27.4 (cyclohexene 4CH_2_), 24.9 (CH_3_), 51.7 (triazine CH_2_), 115.6, 116.3 (2CN), 118.3, 118.9, 119.2, 121.6, 124.6, 133.2, 136.5, 136.9 139.4 (thiophene C, C_6_H_5_, C_6_H_4_), 156.1, 163. 3, 164.6 (3 C=N). *Anal.* Calcd. for C_27_H_24_N_8_S (492.60): C, 65.83; H, 4.91; N, 22.75; S, 6.51%. Found: C, 65.96; H, 5.24; N, 22.55; S, 6.79%.

*Ethyl 1-phenylamino-6-(4-methylphenyl)-3-(α(2-hydrazo-4,5,6,7-tetrahydrobenzo-[b]thiophen-3-carboxylate)acetonitrilo)-1,2,4-triazine* (**10f**). Pale reddish brown crystals (from ethanol). Yield: 0.40 g (59%), m.p. 150–160 °C; IR (KBr) υ/cm^−1^: 3400–3287 (2NH), 2976, 2934, 2860 (2CH_3_, CH_2_), 2213 (CN), 1711 (C=O); ^1^H-NMR δ: 1.07–1.92 (m, 8H, cyclohexene), 2.51 (s, 3H, CH_3_), 2.77 (t, 3H, J = 6.89 Hz, CH_3_), 3.36 (s, 2H, CH_2_), 4.24 (q, 2H, J = 6.89 Hz, CH_2_), 6.50–8.08 (m, 9H, C_6_H_5_, C_6_H_4_), 9.88, 10.30 (2s, 2H, 2NH); ^13^C-NMR: 14.3, 24.8 (2 CH_3_), 23.0, 23.3, 25.1, 27.8 (cyclohexene 4CH_2_), 55.54 (triazine CH_2_), 60.8 (ester CH_2_), 128.7, 129.2, 129.7, 132.0, 136.5, 138.9, 140.8 (C_6_H_5_, thiophene C), 115.8, 116.5 (2CN), 155.2, 158.0, 163.3 (3 C=N), 165.6 (CO); Anal. Calcd. for C_29_H_29_N_7_O_2_S (539.65): C, 64.54; H, 5.42; N, 18.17; S, 5.94%. Found: C, 64.68; H, 5.23; N, 17.97; S, 6.20%.

### 3.6. General Procedure for the Synthesis of ***12a*** and ***12b***

To a solution of compound **6a **(0.47 g, 1.91 × 10^−3^ mol) in ethanol (20 mL) containing triethylamine (0.5 mL), either malononitrile (0.12 g, 1.91 × 10^−3^ mol) or ethyl cyanoacetate (0.21 g, 1.91 × 10^−3^ mol) was added. The reaction mixture was heated under reflux for 3 hrs then poured onto a beaker containing an ice/water mixture and a few drops of hydrochloric acid. The solid product formed was collected by filtration and dried.

*3-Cyano-4,6-diamino-2-oxo-1-imino-(4-methyl-ω-hydrazinoaceto-phenonylidieno)pyridine *(**12a**). Brown crystals (from ethanol). Yield: 0.38 g (64%), m.p. 158 °C; IR (KBr) υ/cm^−1^: 3500–3227 (3NH_2_, NH), 3028 (CH aromatic), 2974 (CH_3_), 2919 (CH_2_), 2201 (CN), 1685 (C=O), 1600 (C=C); ^1^H-NMR δ: 2.51 (s, 3H, CH_3_), 3.38 (s, 2H, CH_2_), 4.38, 4.79, 5.30 (3s, 6H, 3NH_2_), 7.05–8.19 (m, 5H, pyridine H-3, C_6_H_4_), 10.82 (s, 1H, NH); ^13^C-NMR: 23.8 (CH_3_), 51.7 (CH_2_), 80.9, 89.5, 114.8, 125.6 (pyridine C), 117.9 (CN), 126.2, 128.0, 129.3, 129.6 (C_6_H_5_), 163.5 (C=O), 170.9 (C=N); *Anal.* Calcd. for C_15_H_17_N_7_O (311.34): C, 57.87; H, 5.50; N, 31.49%. Found: C, 57.96; H, 5.48; N, 31.68%.

*4-Amino-3-cyano-6-hydroxy-2-oxo-1-imino(4-methyl-ω-hydrazino-acetophenonylidieno)pyridine *(**12b**)*. *Pale brown crystals (from ethanol).Yield: 0.66 g (111%), m.p. 140 °C; IR (KBr) υ/cm^−1^: 3600–3227 (OH, 2NH_2_, NH), 3028 (CH aromatic), 2974 (CH_3_), 2919 (CH_2_), 2201 (CN), 1685 (C=O), 1600 (C=C); ^1^H-NMR δ: 2.50 (s, 3H, CH_3_), 3.39 (s, 2H, CH_2_), 4.34, 4.77, (2s, 4H, 2NH_2_), 7.05–8.19 (m, 5H, pyridine H-3, C_6_H_4_), 10.84 (s, 1H, NH), 12.64 (s, 1H, OH); ^13^C-NMR: 23.6 (CH_3_), 51.4 (CH_2_), 82.9, 89.5, 116.8, 125.9 (pyridine C), 117.9 (CN), 126.4, 128.0, 129.3, 129.6 (C_6_H_5_), 170.9, 176.5 (2C=N), 164.3 (2C=O); *Anal.* Calcd. for C_15_H_16_N_6_O_2_ (312.33): C, 57.68; H, 5.16; N, 26.90%. Found: C, 57.42; H, 5.09; N, 26.76%.

### 3.7. General Procedure for the Synthesis of ***12c*** and ***12d***

To a solution of compound **6b **(0.60 g, 1.86 × 10^−3^ mol) in ethanol (20 mL) containing triethylamine (0.5 mL), either malononitrile (0.12 g, 1.86 × 10^−3^ mol) or ethyl cyanoacetate (0.21 g, 1.86 × 10^−3^ mol) was added. The reaction mixture was heated under reflux for 3 hrs then poured onto a beaker containing an ice/water mixture and a few drops of hydrochloric acid. The solid product formed was collected by filtration and dried.

*3-Cyano-4,6-diamino-2-oxo-1-imino-(4-methyl-ω-phenylhydrazino-acetophenonylidieno)pyridine *(**12c**) Green crystals (from ethanol). Yield: 0.51 g (70%), m.p. 80 °C; IR (KBr) υ/cm^−1^: 3480–3227 (2NH_2_, 2NH), 3028 (CH aromatic), 2974 (CH_3_), 2919 (CH_2_), 2201 (CN), 1685 (C=O), 1600 (C=C); ^1^H-NMR δ: 2.51 (s, 3H, CH_3_), 3.38 (s, 2H, CH_2_), 4.38, 4.79, (2s, 4H, 2NH_2_), 6.48–8.19 (m, 10H, pyridine H-3, C_6_H_5_, C_6_H_4_), 10.82, 11.20 (2s, 2H, 2NH); ^13^C-NMR: 23.7 (CH_3_), 51.9 (CH_2_), 80.7, 89.3, 114.9, 125.3 (pyridine C), 118.3 (CN), 126.4, 128.0, 129.3, 129.6 (C_6_H_5_), 170.9 (C=N), 162.5 (C=O); *Anal.* Calcd. for C_21_H_21_N_7_O (387.44): C, 65.10; H, 5.46; N, 25.30%. Found: C, 64.21; H, 5.36; N, 25.38%.

*4-Amino-3-cyano-6-hydroxy-2-oxo-1-imino(4-methyl-ω-phenyl-hydrazinoacetophenonylidieno) pyridine *(**12d**)*.* Yellowish green crystals (from ethanol). Yield: 0.58 g (80%)**.** Mp 84–100 °C; IR (KBr) υ/cm^−1^: 3600–3185 (OH, NH_2_, 2NH), 3027 (CH aromatic), 2975 (CH_3_), 2919 (CH_2_), 2206 (CN), 1684 (C=O), 1600 (C=C); ^1^H-NMR δ: 2.50 (s, 3H, CH_3_), 3.39 (s, 2H, CH_2_), 4.34 (s, 2H, NH_2_), 6.21 (s, 1H, pyridine H-3), 7.33–8.70 (m, 9H, C_6_H_5_, C_6_H_4_), 10.84, 11.22 (2s, 2H, 2NH), 12.64 (s, 1H, OH);^ 13^C-NMR: 23.6 (CH_3_), 51.6 (CH_2_), 80.2, 89.5, 114.5, 125.0 (pyridine C), 118.1 (CN), 126.2, 128.2, 129.5, 129.6 (C_6_H_5_), 164.8 (C=O), 170.9 (C=N); *Anal.* Calcd. for C_21_H_20_N_6_O_2_ (388.43): C, 64.93; H, 5.19; N, 21.63%. Found: C, 65.42; H, 5.69; N, 21.13%.

### 3.8. General Procedure for the Synthesis of ***14a*** and ***14b***

To a solution of compound **6a **(0.52 g, 2.12 × 10^−3^ mol) in ethanol (20 mL) containing piperidine (0.5 mL), either acetylacetone (0.21 g, 2.21 × 10^−3^ mol) or ethyl acetoacetate (0.27 g, 2.21 × 10^−3^ mol) was added. The reaction mixture was heated under reflux for 3 hrs then poured onto a beaker containing an ice/water mixture and a few drops of hydrochloric acid. The solid product formed was collected by filtration and dried.

*3-Cyano-4,6-diamethyl-2-oxo-1-imino-(4-methyl-ω-hydrazinoaceto-phenonylidieno)pyridine *(**14a**) Brown crystals (from ethanol). Yield: 0.37 g (56%), m.p. 144 °C; IR (KBr) υ/cm^−1^: 3433–3229 (NH_2_, NH,), 3027 (CH aromatic), 2221 (CN), 1685 (C=O), 1600 (C=C); ^1^H-NMR δ: 2.34, 2.54, 3.04 (3s, 9H, 3CH_3_), 3.39 (s, 2H, CH_2_), 6.21 (s, 1H, pyridine H-3), 6.72–7.45 (m, 4H, C_6_H_4_), 10.84, 11.63 (s, 2H, 2NH); ^13^C-NMR: 16.0, 19.2, 24.3 (3CH_3_), 51.2 (CH_2_), 80.0, 88.3, 115.3, 123.9 (pyridine C), 116.8 (CN), 126.0, 127.9, 128.3, 129.1 (C_6_H_5_), 164.9 (C=O), 173.6 (C=N); Anal. Calcd. for C_17_H_19_N_5_O (309.37): C, 66.00; H, 6.19; N, 22.63%; found: C, 66.29; H, 6.23; N, 23.53%.

*3-Cyano-6-hydroxy-4-methyl-2-oxo-1-imino(4-methyl-ω-hydrazino-acetophenonylidieno)pyridine *(**14b**)*.* Brown crystals (from ethanol). Yield: 0.38 g (57%), m.p. 136 °C; IR (KBr) υ/cm^−1^: 3549–3321 (OH, NH_2_, NH), 3027 (CH aromatic), 2213 (CN), 1680 (C=O), 1600 (C=C); ^1^H-NMR δ: 2.53, 3.13 (3s, 9H, 3CH_3_), 3.39 (s, 2H, CH_2_), 6.22 (s, 1H, pyridine H-3), 6.73–7.39 (m, 4H, C_6_H_4_), 10.85, 11.33 (s, 2H, 2NH), 12.21 (s, 1H, OH); ^13^C-NMR: 16.1, 19.0, 24.3 (3CH_3_), 51.3 (CH_2_), 80.2, 88.3, 115.3, 152.1 (pyridine C), 116.6 (CN), 126.3, 127.6, 128.3, 129.1 (C_6_H_5_), 165.3 (C=O), 173.8 (C=N); *Anal*. Calcd. for C_16_H_17_N_5_O_2_ (311.34): C, 61.44; H, 5.50; N, 22.49%; found: C, 61.23; H, 5.67; N, 22.30%.

### 3.9. General Procedure for the Synthesis of ***14c*** and ***14d***

To a solution of compound **6b **(0.60 g, 1.86 × 10^−3^ mol) in ethanol (20 mL) containing piperidine (0.5 mL), either acetylacetone (0.18 g, 1.86 × 10^−3^ mol) or ethyl acetoacetate (0.24 g, 1.86 × 10^−3^ mol) was added. The reaction mixture was heated under reflux for 3 hrs then poured onto a beaker containing an ice/water mixture and a few drops of hydrochloric acid. The solid product formed was collected by filtration and dried.

*3-Cyano-4,6-diamethyl-2-oxo-1-imino-(4-methyl-ω-phenylhydrazino-acetophenonylidieno)pyridine *(**14c**) Yellowish brown crystals (from ethanol). Yield: 0.61 g (85%), m.p. 100 °C; IR (KBr) υ/cm^−1^: 3450–3229 (2NH), 3027 (CH aromatic), 2213 (CN), 1680 (C=O), 1600 (C=C); ^1^H-NMR δ: 2.34, 2.51, 3.01 (3s, 9H, 3CH_3_), 3.37 (s, 2H, CH_2_), 6.21 (s, 1H, pyridine H-3), 6.50–8.41 (m, 9H, C_6_H_5_, C_6_H_4_), 10.82, 12.63 (s, 2H, 2NH); ^13^C-NMR: 16.3, 19.1, 24.3 (3CH_3_), 51.5 (CH_2_), 80.3, 88.6, 115.0, 127.7, 155.8 (pyridine C), 116.5 (CN), 118.7, 119.0, 120.8, 122.5, 126.0, 127.8, 128.7, 129.8 (C_6_H_5_, C_6_H_4_), , 166.2 (C=O), 173.6 (C=N); *Anal*. Calcd. for C_23_H_23_N_5_O (385.47): C, 71.66; H, 6.01; N, 18.16%; found: C, 71.86; H, 5.98; N, 17.99%.

*3-Cyano-6-hydroxy-4-methyl-2-oxo-1-imino(4-methyl-ω-phenyl-hydrazinoacetophenonylidieno)-pyridine *(**14d**)*. *Yellowish brown crystals (from ethanol). Yield: 0.48 g (66%). m.p. 96 °C; IR (KBr) υ/cm^−1^: 3600–3220 (OH, 2NH), 3027 (CH aromatic), 2211 (CN), 1682 (C=O), 1600 (C=C); ^1^H-NMR δ: 2.51, 3.00 (2s, 6H, 2CH_3_), 3.40 (s, 2H, CH_2_), 6.54 (s, 1H, pyridine H-3), 7.34–8.40 (m, 9H, C_6_H_5_, C_6_H_4_), 9.62, 10.82 (2s, 2H, 2NH), 12.65 (s, 1H, OH); ^13^C-NMR: 16.3, 19.4, 24.6 (3CH_3_), 51.5 (CH_2_), 80.8, 88.4, 116.5, 155.4 (pyridine C), 116.5 (CN), 118.8, 119.0, 120.3, 121.7, 126.0, 127.8, 128.9, 129.0 (C_6_H_5_, C_6_H_4_), , 166.4 (C=O), 173.9 (C=N); *Anal*. Calcd. for C_22_H_21_N_5_O_2_ (387.44): C, 68.20; H, 5.46; N, 18.07%; Found: C, 68.49; H, 5.84; N, 17.79%.

*2-Acetonitrilo-5-(4-methylphenyl)-1,3,4-oxadiazine* (**15**)*.* A solution of compound **3** (1.00 g, 3.39 × 10^−3^ mol) in sodium ethoxide (50 mL) was heated under reflux for 3 hrs then poured onto a beaker containing an ice/water mixture and a few drops of hydrochloric acid. The formed solid product was collected by filtration and dried to give pale brown crystals (from ethanol). Yield 0.39 g (54%), m.p. 190–193 °C; IR (KBr) υ/cm^−1^: 3028 (CH aromatic), 2998 (CH_3_), 2922, 2854 (2CH_2_), 2210 (CN), 1673 (C=N), 1609 (C=C); ^1^H-NMR δ: 2.51 (s, 3H, CH_3_), 3.35, 4.42 (2s, 4H, 2CH_2_), 7.00–8.40 (m, 4H, C_6_H_4_); ^13^C-NMR: 24.4 (CH_3_), 20.3 (CH_2_), 64.3 (oxadiazine CH_2_), 116.9 (CN), 126.0, 127.8, 128.9, 129.0, 133.1 (C_6_H_5_), 170.5 (C=N); *Anal*. Calcd. for C_12_H_11_N_3_O (213.24): C, 67.59; H, 5.19; N, 19.70%; found: C, 67.70; H, 5.38; N, 19.88%.

*2-(α-Benzalacetonitrilo)-5-(4-methylphenyl)-1,3,4-oxadiazine hydrochloride *(**16**)*.* To a solution of compound **3** (2.00 g, 6.79 × 10^−3^ mol) in ethanol (30 mL) containing piperidine (0.5 mL), benzaldehyde (0.72 g, 6.79 × 10^−3^ mol) was added. The reaction mixture was heated under reflux for 3 hrs then poured onto a beaker containing an ice/water mixture with a few drops of hydrochloric acid. The formed solid product was collected by filtration and dried to afford brown crystals (from ethanol). Yield: 1.35 g (58%), m.p. 100 °C; IR (KBr) υ/cm^−1^: 3050 (CH aromatic), 3029 (CH_3_), 2921 (CH_2_), 2830 (CH), 2216 (CN), 1604 (C=C); ^1^H-NMR δ: 2.51 (s, 3H, CH_3_), 4.22 (s, 2H, CH_2_), 5.16 (s, 1H, =CH), 7.35–8.02 (m, 9H, C_6_H_5_, C_6_H_4_); ^13^C-NMR: 24.6 (CH_3_), 63.8 (oxadiazine CH_2_), 116.7 (CN), 118.2 (CH=C), 120.4, 121.9, 124.5, 126.2, 127.4, 127.9, 129.2, 133.9 (C_6_H_4_, C_6_H_5_), 144.2 (CH=C), 171.5, 173.9 (2C=N); *Anal*. Calcd. for C_19_H_15_N_3_O (301.34): C, 75.73; H, 5.02; N, 13.94%; found: C, 75.52; H, 4.91; N, 14.31%.

*2-(α-Phenylhydrazoacetonitrilo)-5-(4-methylphenyl)1,3,4-oxadiazine *(**17**)*.* To a cold solution (0–5 °C) of compound **15 **(1.00 g, 3.39 × 10^−3^ mol), in ethanol (50 mL) was added with continuous stirring benzenediazonium chloride (3.39 × 10^−3^ mol) [which was prepared by dissolving sodium nitrite (0.35 g, 5.09 × 10^−3^ mol) in water, 2 mL was added to a cold solution of aniline (0.31 g, 3.39 × 10^−3^ mol) containing the appropriate amount of hydrochloric acid and with continuous stirring]. The solid product formed after adjusting the pH 6 and stirring was collected by filtration and dried to give reddish brown crystals (from ethanol and dimethylformamide). Yield: 0.96 g (89%), m.p. 130 °C; IR (KBr) υ/cm^−1^: 3300–3215 (NH), 3057 (CH aromatic), 3030 (CH_3_), 2920 (CH_2_), 2219 (CN), 1679 (C=N), 1603 (C=C); ^1^H-NMR δ: 2.52 (s, 3H, CH_3_), 3.38 (s, 2H, CH_2_), 7.21–8.82 (m, 9H, C_6_H_5_, C_6_H_4_), 10.22 (s, 1H, NH). ). ^13^C-NMR: 24.3 (CH_3_), 63.0 (oxadiazine CH_2_), 116.5 (CN), 121.5, 121.9, 124.0, 123.5, 126.7, 127.0, 127.6, 129.2, 133.0 (C_6_H_4_, C_6_H_5_), 166.9, 170.5, 173.9 (3C=N). *Anal*. Calcd. for C_18_H_15_N_5_O (317.35): C, 68.12; H, 4.76; N, 22.06%; found: C, 67.92; H, 4.91; N, 21.25%.

## 4. Conclusions

In this work cyanoacetyl hydrazine (*1*) reacted with α-haloketone **2** to afford the α-bromohydrazide-hydrazone derivative **3**. The latter compound was used in a series of heterocyclic transformations to give compounds that were tested as antitumor agents. The 2-hydroxypyridine derivatives **14b** showed the highest inhibitory activity.

## References

[1] Bharti S.K., Nath G., Tilak R., Singh S.K. (2010). Synthesis, anti-bacterial and anti-fungal activities of some novel Schiff bases containing 2,4-disubstituted thiazole ring. Eur. J. Med. Chem..

[2] Loncle C., Brunel J.M., Vidal N., Dherbomez M., Letourneux Y. (2004). Synthesis and antifungal activity of cholesterol-hydrazone derivatives. Eur. J. Med. Chem..

[3] Garoufalias S.P., Pouli N., Marakos P., Ladas A.C. (2002). Synthesis antimicrobial and antifungal activity of some new 3-substituted derivatives of 4-(2,4-dichlorophenyl)-5-adamantyl-1*H*-1,2,4-triazole. Farmaco.

[4] Vicini P., Zani F., Cozzini P., Doytchinova I. (2002). Hydrazones of 1,2-benzisothiazole hydrazides: synthesis, antimicrobial activity and QSAR investigations. Eur. J. Med. Chem..

[5] Popp F.D. (1989). Potential anticonvulsant. XII. anticonvulsant activity of some aldehyde derivatives. Eur. J. Med. Chem..

[6] Sridhar S.K., Pandeya S.N., Stables J.P., Atmakuru R. (2002). Anticonvulsant activity of hydrazones, Schiff and Mannich bases of isatin derivatives. Eur. J. Pharm. Sci..

[7] Küçükgüzel S.G., Mazi A., Sahin F., Öztürk S., Stables J.P. (2003). Synthesis and biological activities of diflunisal hydrazide–hydrazones. Eur. J. Med. Chem..

[8] Todeschini A.R., Miranda A.L.P., Silva K.C.M., Parrini S.C., Barreiro E. (1998). Synthesis and evaluation of analgesic, antiinflammatory and antiplatelet properties of new 2-pyridylarylhydrazone derivatives. Eur. J. Med. Chem..

[9] Gaston M.A., Dias L.R.S., Freitas A.C.C., Miranda A.L.P., Barreiro E. (1996). Synthesis and Analgesic Properties of New 4-Arylhydrazone 1H-Pyrazolo[3,4-b] Pyridine Derivatives. J. Pharm. Acta Helv..

[10] Melnyk P., Leroux V., Sergheraert C., Grellier P. (2006). Design, synthesis and *in vitro* antimalarial of an acylhydrazone library. Bioorg. Med. Chem. Lett..

[11] Küçükgüzel S.G., Rollas S., Kiraz M. (1999). Synthesis and antimycobacterial activity of some coupling products from 4-aminobenzoic acid hydrazones. Eur. J. Med. Chem..

[12] Kaymakçıoğlu B.K., Rollas S. (2002). Synthesis, characterization and evaluation of antituberculosis activity of some hydrazones. Farmaco.

[13] Terzioglu N., Gursoy A. (2003). Synthesis and anticancer evaluation of some new hydrazone derivatives of 2,6-dimethylimidazo[2,1-*b*][1,3,4]thiadiazole-5-carbohydrazide. Eur. J. Med. Chem..

[14] Sondhi S.M., Dinodia M., Kumar A. (2006). Synthesis, anti-inflammatory and analgesic activity evaluation of some amidine and hydrazone derivatives. Bioorg. Med. Chem..

[15] Boga C., Fiume L., Baglioni M., Bertucci C., Farina C., Kratz F., Manerba M., Naldi M., Stefano G. (2009). Characterisation of the conjugate of the (6-maleimidocaproyl)hydrazone derivative of doxorubicin with lactosaminated human albumin by ^13^C NMR spectroscopy. Eur. J. Pharm. Sci..

[16] Garnett M.C. (2001). Targeted drug conjugates: principles and progress. Adv. Drug Deliv. Rev..

[17] Rodrigues P.C., Scheuermann K., Stockmar C., Maier G., Fiebig H., Unger C., Mülhaupt R., Kratz F. (2003). Synthesis and *In vitro *efficacy of acid-Sensitive poly(ethylene glycol) paclitaxel conjugates. Bioorg. Med. Chem. Lett..

[18] El-Sabbagh O.I., Rady H.M. (2009). Synthesis of new acridines and hydrazones derived from cyclic β-diketone for cytotoxic and antiviral evaluation. Eur. J. Med. Chem..

[19] Krakovicova H., Etrych T., Ulbrich K. (2009). HPMA-based polymer conjugates with drug combination. Eur. J. Pharm. Sci..

[20] Zhang H.Z., Drewe J., Tseng B., Kasibhatla S., Cai S.X. (2004). Discovery and SAR of indole-2-carboxylic acid benzylidene-hydrazides as a new series of potent apoptosis inducers using a cell-based HTS assay. Bioorg. Med. Chem..

[21] Vicini P., Incerti M.I., Colla P.L., Loddo R. (2009). Anti-HIV evaluation of benzo[*d*]isothiazole hydrazones. Eur. J. Med. Chem..

[22] Gürsoy E., Güzeldemirci N.U. (2007). Synthesis and primary cytotoxicity evaluation of new imidazo[2,1-*b*]thiazole derivatives. Eur. J. Med. Chem..

[23] Hao J.J., Xu Y., Geng C., Liu W.Y., Wang E., Gong Z., Ulbrich N. (1998). Purification of α-Sarcin and an Antifungal Protein from Aspergillus giganteusby Blue Sepharose CL-6B Affinity Chromatography. Protein Expr. Purif..

[24] Ke S.Y., Qian X.H., Liu F.Y., Wang N., Yang Q., Zhong L. (2009). Novel 4*H*-1,3,4-oxadiazin-5(6*H*)-ones with hydrophobic and long alkyl chains: Design, synthesis, and bioactive diversity on inhibition of monoamine oxidase, chitin biosynthesis and tumor cell. Eur. J. Med. Chem..

